# H-Type Hypertension among Black South Africans and the Relationship between Homocysteine, Its Genetic Determinants and Estimates of Vascular Function

**DOI:** 10.3390/jcdd9120447

**Published:** 2022-12-09

**Authors:** Jacomina P. du Plessis, Leandi Lammertyn, Aletta E. Schutte, Cornelie Nienaber-Rousseau

**Affiliations:** 1 Centre of Excellence for Nutrition, North-West University, Potchefstroom 2520, South Africa; 2 Hypertension in Africa Research Team, North-West University, Potchefstroom 2520, South Africa; 3 Medical Research Council Unit for Hypertension and Cardiovascular Disease, North-West University, Potchefstroom Campus, Potchefstroom 2520, South Africa; 4 School of Population Health, University of New South Wales, The George Institute of Global Health, Sydney, NSW 2000, Australia

**Keywords:** blood pressure management, endothelial function, endothelial structure, H-type hypertension, hyperhomocysteinemia, *MTHFR* C677T, sub-Saharan Africa, vascular inflammation

## Abstract

Elevated homocysteine (Hcy) increases cardiovascular disease (CVD) risk. Our objective was to emphasize Hcy’s contribution in hypertension and CVD management by determining H-type hypertension (hypertension with Hcy ≥ 10 µmol/L) and associations between Hcy, blood pressure (BP) and estimates of vascular function among Black South Africans. We included 1995 adults (63% female). Plasma Hcy and cardiovascular measures (systolic and diastolic BP (SBP, DBP), pulse pressure, heart rate (HR), carotid-radialis pulse wave velocity (cr-PWV), intercellular adhesion molecule-1 (ICAM-1) and vascular cell adhesion molecule-1) were quantified. Five Hcy-related polymorphisms (*cystathionine β-synthase* (*CBS* 844ins68, T833C, G9276A); *methylenetetrahydrofolate reductase* (*MTHFR* C677T) and *methionine synthase* (*MTR* A2756G)) were genotyped. Hcy was >10 µmol/L in 41% (*n* = 762), and of the 47% (*n* = 951) hypertensives, 45% (*n* = 425) presented with H-type. Hcy was higher in hypertensives vs. normotensives (9.86 vs. 8.78 µmol/L, *p* < 0.0001, effect size 0.56) and correlated positively with SBP, DBP, cr-PWV and ICAM-1 (*r* > 0.19, *p* < 0.0001). Over Hcy quartiles, SBP, DBP, HR, cr-PWV and ICAM-1 increased progressively (all *p*-trends ≤ 0.001). In multiple regression models, Hcy contributed to the variance of SBP, DBP, HR, cr-PWV and ICAM-1. H-type hypertensives also had the lowest *MTHFR* 677 CC frequency (*p* = 0.03). Hcy is positively and independently associated with markers of vascular function and raised BP.

## 1. Introduction

Homocysteine (Hcy) is a nonproteinogenic, nonessential, sulfhydryl-containing amino acid. When Hcy is elevated, it conveys an independent risk toward hypertension [1], with Mendelian randomization evidence alluding to a causal relationship [2]. Observations made in the US National Health and Nutrition Examination Survey revealed that hypertensive men and women with Hcy concentrations below 10 µmol/L were 5.4 and 7.1 times more likely to suffer a stroke than normotensives, respectively. However, a combination of both hypertension and Hcy elevated above 10 µmol/L increased the risk substantially to 12.0 and 17.3 times for men and women, respectively [3]. Consequently, hypertension and hyperhomocysteinemia (HHcy) are independent risk factors of stroke, and the combination of both—that is, H-type hypertension—is a major risk factor of vascular diseases [3,4]. H-type hypertension is defined as essential hypertension combined with circulating Hcy greater than 10 µmol/L. Despite its risk to health, however, H-type hypertension has not been investigated in continental African populations [5], who currently demonstrate rapidly increasing hypertension prevalence rates [6,7]. The benefits of reducing Hcy concentrations are unjustifiably underestimated in treatment-resistant hypertension [8].

Because the etiology of essential hypertension has not been fully explained and understood, all factors that could contribute to the condition—including the influence of Hcy-related genes as well as environmental factors and their interactions—should be scrutinized further, some aspects of which we report here. Single genetic polymorphisms within the *methylenetetrahydrofolate reductase* (*MTHFR*) gene, such as the cytosine replacement at position 677 by a thymine (C677T), and other genes coding for proteins involved in Hcy’s metabolism can alter the amino acid’s concentrations [9]. A large-scale meta-analysis reported on the *MTHFR* C677T genotype’s association with hypertension and the homozygote TT genotype conferring an increased risk [2]. However, a follow-up meta-analysis with false-positive report probability interrogations found that the *MTHFR* C677T polymorphism’s relationship was not robust enough [5]. The results of genetic studies are equivocal; this might be because of the variations in the roles played by genotypes in the different ethnic studies reported [2]. If so, it makes it desirable to conduct research on a wider range of ethnic groups in order to establish to what extent Hcy and its genetic markers could influence the high prevalence of hypertension observed in, for example, sub-Saharan Africans [6]. We hope this study will help to re-emphasize Hcy’s contribution in the integral multimodal approach of hypertension and related cardiovascular disease (CVD) management. Consequently, we determined, for the first time, the prevalence of H-type hypertension among Black South African adults and the relationship between plasma Hcy—and its genetic determinants—and blood pressure (BP), as well as estimates of vascular function.

## 2. Materials and Methods

### 2.1. Study Design and Participants

**Figure 1:**
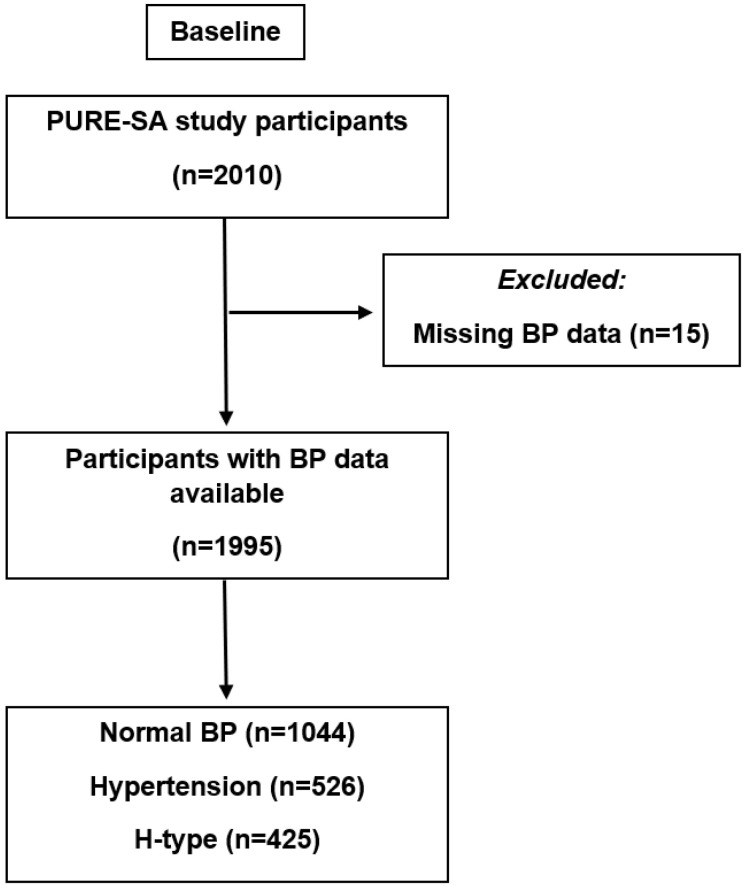
Study design flow chart.

For this cross-sectional investigation, we analyzed the baseline data of 2010 randomly selected participants from the South African arm of the Prospective Urban and Rural Epidemiology (PURE–SA) study. The screening period involved visiting 6000 households in two urbanization strata (rural and urban) within the North West province of South Africa, from which eligible participants were identified as apparently healthy Black South African men and women over the age of 35 years without acute medical conditions. Interested individuals were well informed of all aspects of the study and invited to partake. Written, voluntary, informed consent was required before any measurements were taken, and participants could withdraw from the study at any stage without adverse consequences. The use of hypertension medication was not regarded as a basis for exclusion. For the analytic purposes of this study, volunteers who did not have blood pressure measurements were excluded from the investigation, and analyses proceeded with the remaining 1995 individuals (see Figure 1).

### 2.2. Questionnaires

Standardized questionnaires [10] obtained detailed information from respondents regarding their demographic and lifestyle characteristics by means of face-to-face interviews; these details included age, sex, education level, marital status, medication use, smoking and alcohol consumption.

### 2.3. Anthropometric Measurements

In accordance with the International Society for the Advancement of Kinanthropo-metry (ISAK), anthropometrical measurements were taken by ISAK-trained researchers using calibrated instruments. Measurements included height (IP 1465, Invicta, London, UK) and weight (Precision Health Scale, A&D Company, Tokyo, Japan). Hip and waist circumferences were recorded by means of a Lufkin steel tape (Cooper Tools, Apex, NC, USA), and the measurements were in turn used to calculate body mass index (BMI) (kg/m^2^).

### 2.4. Cardiovascular Marker Measurements

After a 5 min rest period, brachial systolic BP (SBP), diastolic BP (DBP) and heart rate (HR) were measured in duplicate using a validated Omron HEM-757 device (Omron Healthcare, Kyoto, Japan) while volunteers were in a seated position with the measuring (right) arm supported at the heart level. Pulse pressure (PP) was calculated by subtracting DBP from SBP. Carotid radialis pulse wave velocity (cr-PWV) was measured in the supine position and on the left side using the Complior SP device (Artech-Medical, Pantin, France). BP escalating behaviors such as caffeine use (participants were instructed to be fasting, with only water allowed), exercise and smoking were avoided at least 30 min prior to measurements, to ensure accuracy (standardized conditions set by the Joint National Committee on Prevention, Detection, Evaluation and Treatment of High Blood Pressure [11]). Normal BP was identified as BP values <140/90 mmHg. Hypertension was identified in those with a BP ≥ 140 and/or 90 mmHg or who reported anti-hypertensive medication use [12]. H-type hypertension was classified as hypertensive patients with concomitant [Hcy] ≥ 10 µmol/L [13]. Anti-hypertensive medications including thiazide diuretics, calcium channel blockers and renin-angiotensin system inhibitors were grouped together in a categorical variable created for antihypertensive medication use.

### 2.5. Laboratory Biochemical Measurements

A registered nurse sampled blood from the antecubital vein in the mornings between 7 a.m. and 11 a.m. after an overnight fast. Blood samples were centrifuged at 2000× *g* for at least 15 min to obtain serum, plasma and the buffy coat. After being transferred to tubes, the specimens were snap-frozen and then transported on dry ice to be stored at −80 °C.

A recognized pathology firm used an Abbott automated immunoassay analyzer (AxSYM) to quantify Hcy concentrations based on fluorescence polarization immunoassay technology (coefficient of variation (CV) = 4.52%). Both sequential multiple analyzers (Konelab 20i, Thermo Scientific (Vantaa, Finland) and Cobas Integra 400 Plus (Roche, Switzerland)) were used to analyze high-sensitivity C-reactive protein (CRP), gamma-glutamyl transferase (GGT) and blood lipids (triglycerides, total cholesterol (TC) and high-density lipoprotein cholesterol (HDL-C)). The remaining blood lipid, low-density lipoprotein cholesterol (LDL-C) concentration, was calculated using the Friedewald–Levy–Fredrickson formula [14]. Fasting glycated hemoglobin A1c (HbA1c) was measured with a D-10 hemoglobin testing system from Bio-Rad Laboratories (Hercules, CA, USA), and fasting plasma glucose was determined by the hexokinase method of the SynchronR System (Beckman Coulter Co., Fullerton, CA, USA). Serum intercellular adhesion molecule-1 (ICAM-1) and vascular cell adhesion molecule-1 (VCAM-1) levels were determined using sandwich ELISAs (Human sICAM-1 and human sVCAM-1 assay, IBL, Hamburg, Germany).

Genomic DNA was extracted from peripheral blood leukocytes using the commercially available QIAGEN^®^, Flexigene^®^ DNA extraction kits (QIAGEN^®^ Valencia, CA, USA; catalogue number 51 206). Concentrations were determined with the NanoDrop™ spectrophotometer (ND-1000, Wilmington, DE, USA), and polymerase chain reaction coupled with restriction fragment length polymorphism methods enabled the identification of individual genotypes, as described elsewhere [9]. The five Hcy-related single nucleotide polymorphisms (SNPs) genotyped were *cystathionine β-synthase* (*CBS* 844ins68, T833C, G9276A), *methylenetetrahydrofolate reductase* (*MTHFR* C677T) and *methionine synthase* (*MTR* A2756G).

### 2.6. Statistical Analysis

The data were statistically analyzed using the following software packages: Statistica version 14.0 (TIBCO Software Inc., Tulsa, OK, USA) and R statistical software version 4.2.0 (R Core Team; R: A language and environment for statistical computing. R Foundation for Statistical Computing, Vienna, Austria; 2020; URL https://www.r-project.org/; accessed 18 May 2022). Data were tested for normality. Because the data deviated from the normal distribution, as determined by the Shapiro–Wilk W-test and the Kolmogorov–Smirnov tests, descriptive statistics were expressed as medians with interquartile ranges. To determine statistically significant differences in continuous variables, the Kruskal–Wallis ANOVA among three independent sub-groups (normal BP, hypertension and H-type) and the Mann–Whitney U test between two independent groups (normal BP vs. hypertension, normal BP vs. H-type hypertension and hypertension vs. H-type hypertension) were used. Practical significance was determined for differences using Cohen’s effect sizes. For categorical variables, the Pearson chi-squared test was used to detect statistically significant differences and Cramer’s V effect sizes to observe practical significance. Both normal and partial Spearman rank correlations (adjusting for age, sex, BMI and GGT for all analyses and additionally for mean arterial pressure in cr-PWV) were computed to establish the relationship between Hcy and related variables.

Participants were divided into quartiles according to their [Hcy]: <7.44 µmol/L (quartile 1), between ≥7.45 and <9.17 µmol/L (quartile 2), between ≥9.18 and <12.04 µmol/L (quartile 3) and ≥12.05 µmol/L (quartile 4). Differences among the CVD variables and Hcy quartiles were determined using general linear models (GLMs), adjusting for age, sex, BMI and GGT. Where significant differences between groups were indicated, we followed this with a Tukey post hoc test.

Genotype counts and frequencies were determined for the whole group and sub-groups: normal BP, hypertension and H-type hypertension. The Phi correlation coefficient measured the strength of associations between variables, and the chi-squared test was used to determine the significance between groups.

The relationships between BP as well as related cardiovascular variables and Hcy were explored by regression analyses. For regression analyses, the parametric and logarithmically transformed data were used to determine the practical significance of one unit increase in Hcy. For all analyses, a *p* value < 0.05 was regarded as statistically significant.

## 3. Results

The demographic characteristics of the study population are shown in Table 1. The median Hcy and SBP/DBP values were as follows: for all (9.18 µmol/L, 130/87 mmHg), those with a normal BP (8.78 µmol/L, 117/78 mmHg), hypertensives (9.86 µmol/L, 148/97 mmHg), and H-type hypertensives (12.8 µmol/L, 135/89 mmHg). There were no cases of severe HHcy (>100 µmol/L). However, 759 (41%) individuals presented with Hcy >10 µmol/L, 469 (25%) presented with mild Hcy >12 µmol/L and 207 (11%) presented with moderate HHcy >15 µmol/L.

Almost half of the participants (951 (48%)) were hypertensive, and 425 (45%) of those with hypertension presented with H-type hypertension. Of those with Hcy >10 µmol/L, 56% presented with hypertension compared with those with lower Hcy (*p* < 0.0001). Only 262 (13%) participants reported using anti-hypertension medication, of whom 180 (69%) were still hypertensive, including 79 (44%) participants with H-type hypertension.

Statistically significant differences were observed among the BP sub-groups (Table 1 and Table 2). Noteworthy effect sizes were also observed for Hcy and triglycerides (moderate effect sizes) and HbA1c and PP (large effect sizes) in the normal BP vs. hypertension sub-groups. Between the normal BP and H-type hypertension sub-groups, age had a moderate effect size, whereas Hcy and PP had large effect sizes. Hcy attained a large effect size, and ICAM-1 attained a moderate effect size when BP groups of hypertension vs. H-type hypertension were compared. The categorical characteristics such as sex, urbanization and HIV status differed between BP sub-groups, although the effect sizes were small.

In Table 3, Hcy correlated with age, HDL-C, GGT, SBP, cr-PWV and ICAM-1 (*r* ≥ 0.19), but after adjustment for age, sex, BMI and GGT (cr-PWV was additionally adjusted for mean arterial pressure), only correlations with age, HDL-C and GGT remained (*r* ≥ 0.19). Hcy’s relationship with HDL-C and GGT is discussed in detail elsewhere (Du Plessis JP et al. [15] and Nienaber-Rousseau C et al. [16], respectively).

In Table 4, post hoc tests exhibited differences over Hcy quartiles for most of the cardiovascular markers after adjusting for age, sex, BMI and GGT. With ascending Hcy quartiles, SBP, DBP, HR, cr-PWV, ICAM-1 and HDL-C levels increased, with the highest levels reported in the last Hcy quartile. Two blood lipids, TC and LDL-C, were higher in the second Hcy quartile than they were in the other quartiles. Because of the low reporting rate and the high resistance to anti-hypertensive treatment, participants reporting anti-hypertensive medication use were not excluded from analyses; however, additional sensitivity analyses were performed, adjusting for anti-hypertensive medication use (Table 3 and Table 4).

Hcy contributed to the variance of SBP, DBP, HR, cr-PWV and ICAM-1, with the highest contribution in DBP and HR controlling for age, sex, BMI and GGT. With one higher unit of Hcy, DBP and HR increased with 0.12 and 0.16 units, respectively (Table 5).

Genotype distributions were as predicted without deviating from the Hardy–Weinberg equilibrium (*p* > 0.05) (Table 6). The lowest frequency of the *MTHFR* 677CC genotype and, in turn, the highest T allele prevalence were observed in those with H-type hypertension compared to the other BP sub-groups (*p* = 0.03).

## 4. Discussion

Here, we report, for the first time, an H-type hypertension prevalence of 23% among all participants and a 45% prevalence among those with hypertension in a relatively large sample of Black South Africans recruited from both rural and urban communities. We observed that BP related positively and independently to Hcy, which may be due to the adverse effects of Hcy on the measures of vascular structure and function reported here. To complement our investigation, we report on Hcy-related polymorphisms and conclude that, of those considered, the prevalence of the *MTHFR* 677 T allele was greater in those presenting with H-type hypertension.

### 4.1. Homocysteine and Blood Pressure—H-Type Hypertension

Approximately half of the participants (aged 51.0 ± 7 years) were hypertensive, with a positive relationship observed between Hcy and BP; however, these correlations diminished after adjustments. Nonetheless, both SBP and DBP values rose progressively over increasing Hcy quartiles, and DBP even exceeded the upper BP range (<90 mmHg) in the highest Hcy quartile, regardless of adjustments. These findings, together with the large effect sizes observed between BP sub-groups, confirm that Hcy plays a meaningful role in both SBP and DBP levels, irrespective of age, sex, BMI and GGT, in Black South African adults. Previous studies reported a similar association between Hcy and BP in adults [17,18] as well as in Black South African adolescents [19].

The participants in the hypertension sub-group also had higher Hcy concentrations, with a concomitant increase in the prevalence of H-type hypertension. The prevalence of H-type hypertension observed here reflects that reported by Towfighi et al. [3], who re-corded a 48% prevalence among American adults, less than the 75% and higher prevalence reported in Chinese adults [20,21], reaffirming the importance of multi-ethnic investigations before evaluating intervention possibilities. The pathogenesis of H-type hypertension still needs further exploration. Because environmental and genetic factors as well as their interactions could contribute to the pathophysiology of H-type hypertension and resistant hypertension, investigations should incorporate both [2].

### 4.2. H-Type Hypertension and Genetic Determinants

Our findings provide an evidence-based reference for H-type hypertension in Black South Africans, with the possibility of the *MTHFR* C677T polymorphism becoming a new marker for the clinical evaluation of H-type hypertension in this population. Our results add to the body of evidence indicating that *MTHFR* C677T could be related to H-type hypertension susceptibility [22]. The frequency of the CC genotype of the *MTHFR* polymorphism, known to have protective qualities against hypertension [2,23], was the lowest in those with H-type hypertension, resulting in the T allele being the most prevalent in this BP sub-group. Those carrying genes predisposing them to hypertension and Hcy-related diseases could benefit from dietary interventions personalized according to their genetic make-up [15].

A recent study reported that patients treated with folic acid doses, individualized according to their *MTHFR* C677T genotype, exhibited reduced BP and Hcy and an improved prothrombotic status in those with H-type hypertension [24]. The *MTHFR* 677T allele causes a decrease in the essential cofactor flavin adenine dinucleotide’s affinity, with riboflavin as a precursor, demonstrating higher BP values and increased hypertension risk. Genome-wide studies [25] and a randomized control trial [26] reported substantial reductions in BP after riboflavin (vitamin B_2_) supplementation, more so than those with anti-hypertensive treatment alone. These studies emphasize an alternative and safe treatment opportunity for resistant hypertension through vitamin supplementation. Identifying genetic risk factors opens the possibility for risk stratification and personalized prevention as well as treatments such as genotype-guided dietary intake and even possible targeted gene therapy opportunities.

### 4.3. Hcy with Vascular Function and Inflammation Markers

As expected, we confirmed positive correlations between Hcy and some preclinical markers of vascular function and inflammation. However, after statistical adjustments, the associations diminished, with only HR remaining. When evaluating the cardiovascular markers over Hcy quartiles, peripheral arterial stiffness (cr-PWV) and vascular inflammation (ICAM-1) exceeded normal reference intervals (as provided by Bia and Zócalo [27] and Rothlein R et al. [28], respectively), corresponding to the greatest Hcy concentration quartile examined.

Elevated Hcy values have been indicated in both arterial stiffness and vascular inflammation, leading to endothelial dysfunction and hypertension development [29]. The postulated mechanisms involved include Hcy interfering with the production of vascular regulating nitric oxide and deregulating the signaling pathway associated with hydrogen sulfide (H_2_S), resulting in endothelial imbalance [29]. A Chinese longitudinal community-based study reported Hcy to be positively associated with central arterial stiffness (carotid femoral-PWV) but not with peripheral arterial stiffness (cr-PWV), as observed in our study [30]. Further research is needed to evaluate the relationship between Hcy and different measures of arterial stiffness in multi-ethnic populations.

Hcy has been associated with vascular inflammation either directly or indirectly via the production of reactive oxygen species [31] and has also been indicated to initiate inflammatory responses within the vascular smooth muscle cells by stimulating CRP production [32]. Moreover, Barroso, Kao [30] reported that, in endothelial cells, the precursor of Hcy, S-adenosylhomocysteine, activates nuclear factor kappa B (NF-κB) and initiates the expression of pro-inflammatory molecules, including interleukin-1, ICAM-1, VCAM-1 and E-selectin. Durga, Van Tits [33], however, reported that a noticeable lowering of elevated Hcy concentrations did not influence inflammatory responses involving CRP, ICAM-1, oxidized LDL-C or autoantibodies against oxidized LDL-C. Further research is needed to clarify Hcy’s role in vascular inflammation, because one of the enzymes in the Hcy transsulfuration pathway, CBS, is a major source of vascular H_2_S, which inhibits vascular inflammation by inhibiting the NF-kB pathway [29].

### 4.4. Treatment and Recommendations

The least invasive and most cost-effective way of potentially manipulating Hcy is through diet and lifestyle changes. Consequently, the relationships between Hcy—and its genetic markers—and markers of vascular function should be further explored to confirm causality in trials where Hcy is lowered. Because African Americans and Asians have a 3–4 times higher risk of angioedema than Whites as a side effect of using anti-hypertensive medicine [11], alternative treatments are therefore necessary for those experiencing adverse pharmacological consequences. The Dietary Approaches to Stop Hypertension (DASH) lifestyle recommends a low-fat diet that is high in fruits, vegetables and low-fat dairy. The DASH lifestyle has been reported to reduce Hcy concentrations in addition to lowering BP [34,35]. Moreover, several other lifestyle factors are known to reduce BP, namely, weight loss, lowering sodium intake, regular physical activity and limiting alcohol consumption [11] and should also be investigated along with diet to lower Hcy and contingent CVD risk.

### 4.5. Limitations, Strengths and Future Studies

The large sample size reported here was critical, as it ensured the means to detect small changes in BP. This study could assess some associations, but, unfortunately, due to its cross-sectional nature, no inferences concerning the causal relationship of high Hcy and hypertension can be made. To examine any possible causal effect of Hcy lowering and BP, we suggest controlled intervention trials. Future research should investigate the interactions between dietary factors and Hcy-related genetic variations, especially the *MTHFR* C677T polymorphism, in relation to BP and markers of vascular structure and function or hard clinical outcomes. Thereafter, dietary supplementation trials stratified for genotypes should be conducted to evaluate their efficacy. Markers such as intima media thickness and carotid femoral-PWV, the “golden standard” measure for arterial stiffness, are not evaluated and should be included in future studies. H-type hypertension is an old concept with a new label, drawing attention to the relationship between Hcy and hypertension. By embracing the new single term, researchers will increase the searchabi-lity and visibility of articles still referring to HHcy and BP separately [36]. The use of the term “H-type hypertension” should be encouraged in future studies of this kind, which should include different ethnicities across their respective geographical regions to broaden the value and understanding of research on H-type hypertension.

## 5. Conclusions

This study has demonstrated a potentiating relationship between Hcy and raised BP, which could lead to safe, tailored prospects for CVD prevention. For example, Hcy can be lowered by lifestyle modifications including dietary supplementation with folate and B-vitamins. Such treatment can ultimately improve BP outcomes and subsequent stroke risk. Patients with elevated Hcy, hypertension or H-type hypertension should be consi-dered as candidates for screening and lifestyle changes. These modifications, together with appropriate supplementation, can forestall hypertension in pre-hypertensives—especially using folic acid and riboflavin in addition to, or as alternatives to, expensive pharmacological medications when they are unavailable, ineffective or induce adverse side effects. Moreover, this study indicates that specific genetic factors may dictate different prevention or treatment strategies. Future investigations should further explore the relationships between determinants of Hcy, especially gene–diet interactions, in relation to BP and markers of the vascular function of clinical outcomes such as stroke, to determine potential conflated associations informing intervention strategies.

## Figures and Tables

**Table 1:** Demographic characteristics of participants over blood pressure sub-groups.

		Median (25th–75th) or *n* (%)				
		Whole Group (*n* = 1995)	Normal BP (*n* = 1044)	Hypertension (*n* = 526)	H-Type Hypertension * (*n* = 425)	Differences Among Groups # *p* Value
Age (years)		48.0 (41.0–56.0)	45.0 (40.0–53.0) ^@^	49.0 (43.0–57.0) ^@^	53.0 (46.0–61.0) ^@^	<0.0001
Sex	Male	743	393 (52.9) ^@^	160 (21.5) ^@^	190 (25.6) ^@^	<0.0001
	Female	1252	651 (52.0) ^@^	366 (29.2) ^@^	235 (18.8) ^@^	
Urbanization level	Urban	996	456 (45.8) ^@^	330 (33.1) ^@^	210 (21.1) ^@^	<0.0001
	Rural	999	588 (58.9) ^@^	196 (19.6) ^@^	215 (21.5) ^@^	
Tobacco use	Current	1033	544 (52.7)	272 (26.3)	217 (21.0)	0.71
	Former	77	39 (50.6)	19 (24.7)	19 (24.7)	
	Never	875	455 (52.0)	231 (26.4)	189 (21.6)	
Anthro markers	BMI (kg/m^2^)	23.0 (19.3–28.9)	22.1 (18.9–27.5) ^@,†^	25.3 (20.2–31.7) ^@,$^	23.1 (19.5–28.8) ^$,†^	<0.0001
	Waist circumference (cm)	77.45 (70.2–87.7)	74.7 (68.8–84.3) ^@^	81.9 (72.5–92.7) ^@^	80.1 (72.2–89.1) ^@^	<0.0001
	Hip circumference (cm)	93.13 (84.8–106)	91.4 (83.8–103) ^@^	97.3 (87.4–111) ^@^	92.2 (85.2–104) ^@^	<0.0001
	Waist-to-hip ratio	0.83 (0.78–0.88)	0.82 (0.77–0.87) ^@^	0.83 (0.78–0.88) ^@^	0.85 (0.81–0.90) ^@^	<0.0001
Biochemical markers	HIV sero-negative	1655	820 (49.5) ^@^	448 (27.1) ^@^	387 (23.4) ^@^	<0.0001
	HIV sero-positive	324	215 (66.4) ^@^	73 (22.5) ^@^	36 (11.1) ^@^	
	HIV status unknown	14	9 (64.3)	4 (28.6)	1 (7.10)	
	TC (mmol/L)	4.82 (4.01–5.87)	4.68 (3.87–5.67) ^@^	5.02 (4.22–6.12) ^@^	4.96 (4.15–6.06) ^@^	<0.0001
	LDL-C (mmol/L)	2.77 (2.07–3.63)	2.73 (2.06–3.52) ^$^	2.91 (2.20–3.82) ^$^	2.77 (1.97–3.67)	0.01
	HDL-C (mmol/L)	1.42 (1.06–1.87)	1.35 (1.01–1.80) ^@^	1.37 (1.05–1.85) ^@^	1.60 (1.18–2.11) ^@^	<0.0001
	Triglycerides (mmol/L)	1.08 (0.82–1.55)	1.01 (0.79–1.40) ^@^	1.18 (0.86–1.70) ^@, $^	1.12 (0.82–1.70) ^$^	<0.0001
	Fasting glucose (mmol/L)	4.80 (4.30–5.30)	4.80 (4.30–5.20) ^†^	4.80 (4.30–5.40)	4.90 (4.40–5.45) ^†^	0.01
	HbA1c (%)	5.50 (5.30–5.80)	5.50 (5.30–5.80) ^†^	5.60 (5.30–5.90) ^†^	5.50 (5.20–5.85)	0.02
	GGT (µkat/L)	46.0 (29.7–88.0)	40.4 (28.0–69.0) ^@,$^	49.5 (30.8–82.3) ^$^	57.4 (33.8–139.8) ^@^	<0.0001
	Hcy (µmol/L)	9.18 (7.50–12.1)	8.78 (7.16–11.0) ^@^	8.00 (6.78–8.99) ^@^	12.8 (11.4–15.9) ^@^	<0.0001
	CRP (mg/L)	3.29 (0.96–9.34)	2.80 (0.73–9.29) ^@^	3.77 (1.39–9.08) ^†^	3.76 (1.16–9.67)	<0.0001
Cardiovascular markers	SBP (mmHg)	130 (116–147)	117 (109–126) ^@^	146 (136–160) ^@^	135 (120–152) ^@^	<0.0001
	DBP (mmHg)	87.0 (78.0–97.0)	78.0 (72.0–84.0) ^@^	97.0 (92.0–105) ^@^	89.0 (80.0–99.0) ^@^	<0.0001
	Pulse pressure (mmHg)	43.0 (35.0–53.0)	39.0 (33.0–45.0) ^@^	50.0 (40.0–61.0) ^@^	45.0 (36.0–56.0) ^@^	<0.0001
	Heart rate (bpm)	72.0 (62.0–84.0)	71.0 (62.0–84.0) ^@^	71.0 (61.0–81.0) ^@^	74.0 (63.0–87.0) ^@^	<0.0001
	cr-PWV (m/s)	10.9 (9.55–12.2)	10.5 (9.17–11.8) ^@^	11.2 (9.98–12.3) ^@,†^	11.2 (10.0–12.8) ^@,†^	<0.0001
	ICAM-1 (ng/mL)	371 (234–507)	377 (247–510)	489 (327–805)	440 (286–548)	0.83
	VCAM-1 (ng/mL)	693 (390–1279)	679 (384–1295)	751 (357–1308)	662 (449–1139)	0.99
Anti-hypertensives use *n* (%)		283	82 (29.0) ^@^	122 (43.1) ^@^	79 (27.9) ^@^	<0.0001

BMI, body mass index; CRP, C-reactive protein; cr-PWV, carotid-radialis pulse wave velocity; DBP, diastolic blood pressure; GGT, gamma glutamyl transferase; Hcy, homocysteine; HDL-C, high-density lipoprotein cholesterol; HbA1c, glycated hemoglobin; HIV, human immunodeficiency virus; HR, heart rate; ICAM-1, intercellular adhesion molecule 1; LDL-C, low-density lipoprotein cholesterol; PP, pulse pressure; SBP, systolic blood pressure; VCAM-1, vascular cell adhesion molecule 1.* H-type hypertension is a subset of those presenting with both hypertension and Hcy > 12.0 µmol/L and has thus been excluded from the hypertension group to form two independent groups. # Kruskal–Wallis ANOVA *p* values for continuous values and Pearson Chi-squared *p* value for categorical values. Post hoc analysis differences indicated with the same symbol; @ denotes *p* > 0.0001, $ denotes *p* < 0.001, † denotes *p* < 0.05.

**Table 2:** Comparison of demographic, anthropometric and biochemical variables between hypertension status sub-groups.

Variables		Normal vs. HTN		Normal vs. H-Type HTN		HTN vs. H-Type HTN *	
		*p* Value	Effect Size	*p* Value	Effect Size	*p* Value	Effect Size
Continuous Variables							
	Age (years)	<0.0001	0.36	<0.0001	0.69	<0.0001	0.33
Anthro markers	BMI (kg/m^2^)	<0.0001	0.40	0.58	0.17	0.001	0.21
	Waist circumference (cm)	<0.0001	0.45	<0.001	0.35	0.16	0.10
	Hip circumference (cm)	<0.0001	0.37	0.55	0.09	<0.0001	0.26
	Waist-to-hip ratio	<0.0001	0.16	<0.0001	0.45	<0.0001	0.27
Biochemical markers	TC (mmol/L)	<0.0001	0.24	<0.01	0.26	0.31	0.01
	LDL-C (mmol/L)	0.04	0.15	0.34	0.03	0.89	0.11
	HDL-C (mmol/L)	<0.0001	0.11	<0.0001	0.37	0.001	0.26
	Triglycerides (mmol/L)	<0.0001	0.79	0.03	0.23	0.23	0.08
	Fasting glucose (mmol/L)	0.001	0.23	0.05	0.13	0.32	0.10
	HbA1c (%)	0.04	0.88	0.41	0.07	0.11	0.15
	GGT (µkat/L)	<0.0001	0.11	<0.0001	0.33	0.03	0.15
	Hcy (µmol/L)	<0.0001	0.56	<0.0001	1.13	<0.0001	1.87
	CRP (mg/L)	<0.01	0.09	0.28	0.05	0.86	0.05
Cardiovascular markers	SBP (mmHg)	<0.0001	2.10	<0.0001	2.42	0.53	0.11
	DBP (mmHg)	<0.0001	2.35	<0.0001	2.38	0.57	0.09
	Pulse pressure (mmHg)	<0.0001	1.00	<0.0001	1.19	0.67	0.08
	Heart rate (bpm)	0.45	0.13	<0.0001	0.22	<0.0001	0.34
	cr-PWV (m/s)	<0.0001	0.33	<0.0001	0.49	0.04	0.17
	ICAM-1 (ng/mL)	0.14	0.02	0.03	0.03	0.21	0.51
	VCAM-1 (ng/mL)	0.30	0.06	0.01	0.14	0.86	0.17
**Categorical Variables**							
Sex	Male/Female	0.01	0.07	0.01	0.07	<0.0001	0.15
Urbanization level	Urban/Rural	<0.0001	0.18	0.05	0.05	<0.0001	0.14
Tobacco use	Current/Former/Never	0.96	0.01	0.40	0.05	0.27	0.06
HIV status	Sero-negative/-positive/Unknown	0.01	0.08	<0.0001	0.16	0.02	0.09
Anti-hypertensives use	Yes/No	<0.0001	0.18	<0.0001	0.21	0.27	0.04

Continuous variables: Mann–Whitney U test for differences between groups and Cohen’s effect sizes. Categorical variables: Pearson’s chi-squared test for differences between groups and Cramer’s V effect sizes. * H-type hypertension is a subset of those presenting with both hypertension and Hcy >12.0 µmol/L and has thus been excluded from the hypertension group to form two independent groups.

**Table 3:** Spearman correlations between Hcy and characteristics of participants.

Variables		Unadjusted		Adjusted		Sensitivity Analysis	
		*r*	*p*	*r*	*p*	*r*	*p*
Age (year)		0.28	<0.0001	0.27	<0.0001	0.27	<0.0001
**Anthropometrical Markers**							
BMI (kg/m^2^)		−0.13	<0.0001	−0.04	0.35	−0.16	<0.0001
Waist circumference (cm)		−0.03	0.24	0.02	0.69	−0.04	0.09
Hip circumference (cm)		−0.14	<0.0001	0.01	0.82	−0.16	<0.0001
Waist-to-hip ratio		0.17	<0.0001	−0.02	0.67	0.17	<0.0001
**Biochemical Markers**							
TC (mmol/L)		0.05	0.02	0.10	0.03	0.05	0.06
LDL-C (mmol/L)		−0.05	0.03	0.01	0.91	−0.07	<0.01
HDL-C (mmol/L)		0.19	<0.0001	0.20	<0.0001	0.20	<0.0001
Triglycerides (mmol/L)		0.001	0.96	−0.06	0.16	−0.02	0.54
Fasting glucose (mmol/L)		0.002	0.92	−0.07	0.10	−0.02	0.34
HbA1c (%)		−0.05	0.02	−0.02	0.64	−0.06	0.01
GGT (µkat/L)		0.24	<0.0001	0.24	<0.0001	0.23	<0.0001
CRP (mg/L)		0.03	0.15	0.01	0.86	0.01	0.60
**Cardiovascular Markers**							
Blood pressure (mmHg)	SBP	0.19	<0.0001	0.07	0.14	0.19	<0.0001
	DBP	0.16	<0.0001	0.08	0.09	0.15	<0.0001
Pulse pressure (mmHg)		0.14	<0.0001	0.01	0.76	0.14	<0.0001
Heart rate (bpm)		0.11	<0.0001	0.12	0.01	0.09	<0.001
cr-PWV (m/s)		0.19	<0.0001	0.04	0.32	0.21	<0.0001
ICAM-1 (ng/mL)		0.23	<0.0001	–0.02	0.68	0.21	<0.0001
VCAM-1 (ng/mL)		0.04	0.35	0.03	0.53	0.05	0.23

In the unadjusted model, the *r* values are for Spearman correlations; in the adjusted model, the *r* values are for Spearman partial correlations adjusted for age, sex, BMI and GGT. cr-PWV was additionally adjusted for mean arterial pressure. Anthropometrical markers were not adjusted for BMI, and the age and GGT variables were not adjusted for age and GGT, respectively. In the sensitivity analysis, individuals using anti-hypertensive medication were excluded from analyses. BMI, body mass index; CRP, C-reactive protein; cr-PWV, carotid-radialis pulse wave velocity; DBP, diastolic blood pressure; GGT, gamma glutamyl transferase; HDL-C, high-density lipoprotein cholesterol; HbA1c, glycated hemoglobin; HR, heart rate; ICAM-1, intercellular adhesion molecule 1; LDL-C, low-density lipoprotein cholesterol; PP, pulse pressure; SBP, systolic blood pressure; VCAM-1, vascular cell adhesion molecule 1.

**Table 4:** Cardiovascular markers across Hcy quartiles.

CVD Marker	Homocysteine (Adjusted Means with 95% CI Determined through GLM)					
	Quartile 1 (*n* = 464) <7.44 µmol/L	Quartile 2 (*n* = 467) ≥7.45 to <9.17 µmol/L	Quartile 3 (*n* = 469) ≥9.18 to <12.04 µmol/L	Quartile 4 (*n* = 467) >12.05 µmol/L	GLM *p* Value	Sensitivity *p* Value
CRP (mg/L)	8.12 (6.97–9.27)	8.17 (7.05–9.29)	8.16 (7.04–9.29)	8.94 (7.78–10.1)	0.73	0.88
Fasting glucose (µmol/L)	5.05 (4.90–5.21)	4.95 (4.81–5.10)	5.01 (4.86–5.15)	4.90 (4.75–5.06)	0.58	0.11
SBP (mmHg)	131 (129–133) ^c,d,f^	133 (131–135) ^c,g^	133 (130–135) ^d,h^	137 (135–139) ^f,g,h^	0.001	0.01
DBP (mmHg)	85.4 (84.1–86.7) ^c,f^	85.9 (84.6–87.2) ^g^	87.5 (86.2–88.8) ^c,d^	90.9 (89.6–92.2) ^f,d,g^	<0.0001	<0.0001
PP (mmHg)	45.6 (44.3–46.9)	46.9 (45.6–48.2)	45.0 (43.8–46.3)	46.1 (44.8–47.4)	0.21	0.37
HR (bpm)	71.9 (70.4–73.3) ^g^	71.6 (70.3–73.0) ^f^	73.3 (71.9–74.7) ^h^	77.5 (76.1–79.0) ^f,g,h^	<0.0001	<0.0001
cr-PWV (m/s)	10.7 (10.5–10.9) ^a,f,g^	10.9 (10.7–11.2) ^a,h^	11.1 (10.9–11.3) ^f,b^	11.3 (11.1–11.5) ^g,h,b^	<0.0001	0.001
ICAM-1 (ng/mL)	335 (310–360) ^a,g^	351 (326–377) ^c^	383 (356–409) ^a^	420 (393–449) ^c,g^	<0.0001	0.001
VCAM-1 (ng/mL)	1053 (853–1254)	1127 (914–1340)	1042 (814–1269)	909 (661–1157)	0.63	0.59
TC (mmol/L)	4.87 (4.74–5.00) ^c^	5.14 (5.02–5.26) ^c^	5.02 (4.89–5.14)	5.04 (4.91–5.17)	0.03	0.07
LDL-C (mmol/L)	2.90 (2.80–3.01)	3.03 (2.93–3.14) ^c^	2.92 (2.82–3.03)	2.80 (2.69–2.91) ^c^	0.001	0.04
HDL-C (mmol/L)	1.38 (1.32–1.44) ^c,d,g^	1.52 (1.46–1.57) ^c,f^	1.52 (1.47–1.58) ^d,e^	1.66 (1.60–1.71) ^g,f,e^	<0.0001	<0.0001
Triglicerides (mmol/L)	1.29 (1.22–1.36)	1.29 (1.23–1.36)	1.26 (1.19–1.33)	1.29 (1.22–1.36)	0.89	0.65

Post hoc test revealed significant differences between the quartiles indicated with letter(s); quartiles with the same symbol differ with the level of significance denoted as follows *p* < 0.05 ^a,b^, ≤0.01 ^c^, <0.001 ^d,e^, <0.0001 ^f,g,h^. GLMs were adjusted for age, sex, BMI and GGT. Additional analyses that excluded individuals using anti-hypertensive medication are reported as sensitivity *p* value. CRP, C-reactive protein; cr-PWV, carotid-radialis pulse wave velocity; DBP, diastolic blood pressure; HDL-C, high-density lipoprotein cholesterol; HR, heart rate; ICAM-1, intercellular adhesion molecule 1; LDL-C, low-density lipoprotein cholesterol; PP, pulse pressure; SBP, systolic blood pressure; TC, total cholesterol; VCAM-1, vascular cell adhesion molecule 1.

**Table 5:** Multivariable-adjusted relationships of cardiovascular measures with Hcy.

Variables	SBP (R^2^ = 0.18)			
	B	SE	β	*p*
Age	0.71	0.05	0.30	<0.0001
Sex	5.34	1.21	0.11	<0.0001
BMI (kg/m^2^)	0.41	0.09	0.12	<0.0001
GGT (μkat/L)	0.004	0.003	0.03	0.13
Hcy (μmol/L)	0.43	0.12	0.08	<0.0001
Anti-hypertensive use	8.92	1.51	0.14	<0.0001
	**DBP (R^2^ = 0.10)**			
	**B**	**SE**	**β**	***p***
Age	0.17	0.03	0.12	<0.0001
Sex	0.21	0.75	0.01	0.79
BMI (kg/m^2^)	0.33	0.05	0.16	<0.0001
GGT (μkat/L)	0.01	0.002	0.09	<0.0001
Hcy (μmol/L)	0.38	0.08	0.12	<0.0001
Anti-hypertensive use	5.13	0.94	0.13	<0.0001
	**PP (R^2^ = 0.19)**			
	**B**	**SE**	**β**	***p***
Age	0.54	0.03	0.37	<0.0001
Sex	5.14	0.74	0.16	<0.0001
BMI (kg/m^2^)	0.08	0.05	0.04	0.11
GGT (μkat/L)	–0.002	0.002	–0.03	0.19
Hcy (μmol/L)	0.04	0.07	0.01	0.57
Anti-hypertensive use	3.80	0.92	0.09	<0.0001
	**HR (R^2^ = 0.10)**			
	**B**	**SE**	**β**	***p***
Age	–0.20	0.04	–0.13	<0.0001
Sex	–8.25	0.83	–0.25	<0.0001
BMI (kg/m^2^)	–0.21	0.06	–0.09	<0.0001
GGT (μkat/L)	0.01	0.002	0.13	<0.0001
Hcy (μmol/L)	0.56	0.08	0.16	<0.0001
Anti-hypertensive use	2.40	1.04	0.06	0.02
	**cr-PWV (R^2^ = 0.15)**			
	**B**	**SE**	**β**	***p***
Age	0.01	0.01	0.05	0.04
Sex	0.77	0.12	0.16	<0.0001
BMI (kg/m^2^)	–0.08	0.01	–0.25	<0.0001
GGT (μkat/L)	0.001	<0.0001	0.05	0.02
Hcy (μmol/L)	0.04	0.01	0.09	<0.0001
Anti-hypertensive use	0.20	0.15	0.03	0.17
	**ICAM-1 (R^2^ = 0.08)**			
	**B**	**SE**	**β**	***p***
Age	2.27	2.04	0.05	0.27
Sex	−5.73	45.9	−0.01	0.90
BMI (kg/m^2^)	−3.34	3.20	−0.05	0.30
GGT (μkat/L)	0.71	0.11	0.28	<0.0001
Hcy (μmol/L)	−11.7	4.58	-0.11	0.01
Anti-hypertensive use	95.1	57.2	0.07	0.10
	**VCAM (R^2^ = 0.01)**			
	**B**	**SE**	**β**	***p***
Age	4.51	5.61	0.04	0.42
Sex	59.11	126	0.02	0.64
BMI (kg/m^2^)	−7.08	8.79	−0.04	0.42
GGT (μkat/L)	0.02	0.29	0.003	0.95
Hcy (μmol/L)	−15.0	12.6	−0.05	0.23
Anti-hypertensive use	−57.0	157	−0.02	0.71

**β**, standardized beta; B, unstandardized beta; BMI, body mass index; DBP, diastolic blood pressure; cr-PWV, carotid-radialis pulse wave velocity; GGT, gamma glutamyl transferase; Hcy, homocys-teine; HR, heart rate; ICAM-1, intercellular adhesion molecule 1; PP, pulse pressure; SBP, systolic blood pressure; SE, standard error; VCAM-1, vascular cell adhesion molecule 1.

**Table 6:** Hcy-related SNPs and their genotype frequencies in population subdivisions for blood pressure.

Gene; SNP ID (rs Number; Location)	Whole Group	Normotensive	Hypertension	H-Type Hypertension
	Genotype Counts (Frequencies %)			
*MTHFR*; C677T Ala222Val (rs1801133; 1:11796321)	CC 1561 (84.0)	821 (83.3)	404 (87.1)	336 (82.4)
	CT 282 (15.2)	156 (15.8)	58 (12.5)	68 (16.7)
	TT 15 (0.80)	9 (0.90)	2 (0.40)	4 (0.90)
	Phi = 0.08			
	Chi-square *p* = 0.03			
*MTR*; A2756G Asp919Gly (rs1805087; 1:236885200)	AA 1181 (63.7)	621 (63.3)	284 (61.2)	276 (67.7)
	AG 583 (31.5)	315 (32.1)	154 (33.2)	114 (27.9)
	GG 89 (4.80)	45 (4.60)	26 (5.60)	18 (4.40)
	Phi = 0.06			
	Chi-square *p* = 0.28			
*CBS*; T833C Ile278Thr (rs5742905; 21:43063074)	TT 984 (52.9)	520 (52.7)	246 (52.9)	218 (53.2)
	TC 740 (39.8)	396 (40.2)	187 (40.2)	157 (38.3)
	CC 137 (7.30)	70 (7.10)	32 (6.90)	35 (8.50)
	Phi = 0.05			
	Chi-square *p* = 0.55			
*CBS*; 844ins68 indel (no rs#)	WT 985 (52.9)	521 (52.8)	245 (52.7)	219 (53.3)
	HT 742 (39.8)	396 (40.2)	188 (40.4)	158 (38.4)
	MT 135 (7.30)	69 (7.00)	32 (6.90)	34 (8.30)
	Phi = 0.04			
	Chi-square *p* = 0.80			
*CBS*; G9276A (novel SNP no rs#; 21:43071860)	GG 966 (51.9)	517 (52.4)	232 (50.0)	217 (52.9)
	GA 750 (40.3)	391 (39.7)	199 (42.9)	160 (39.0)
	AA 144 (7.80)	78 (7.90)	33 (7.10)	33 (8.10)
	Phi = 0.04			
	Chi-square *p* = 0.73			

844ins68, insertion of 68 base pairs at nucleotide position 844; A, adenine (nucleotide); Ala, alanine; Asp, aspartic acid; C, cytosine (nucleotide); *CBS*, *cystathionine β-synthase gene*; G, guanine (nucleotide); Gly, glycine; HT, heterozygous; ID, identity; Ile, isoleucine; indel, insertion/deletion; MT, homozygous insert; MTHFR, *methylenetetrahydrofolate reductase gene*; MTR, *methionine synthase gene*; rs, reference SNP; SNP, single nucleotide polymorphism; T, thymine (nucleotide); Thr, threonine; Val, valine; WT, homozygous non-insert. Genotype information expressed here is for individuals for whom we had genetic and BP data available. * Individuals with H-type hypertension are a subset of those presenting with hypertension for whom we had BP and Hcy data, with Hcy being >10 µmol/L.

## Data Availability

The data analyzed in this study are subject to the following licenses/restrictions: Raw data were generated at the North-West University. Obtained data supporting the findings of this study are available from the PI of the study after obtaining the necessary ethical approval. Due to ethical restrictions and the POPI act, data are not publicly available. Requests to access these datasets should be directed to lanthe.kruger@nwu.ac.za.
